# Robust optical fiber patch-cords for *in vivo* optogenetic experiments in rats

**DOI:** 10.1016/j.mex.2015.05.003

**Published:** 2015-05-18

**Authors:** Ivan Trujillo-Pisanty, Christian Sanio, Nadia Chaudhri, Peter Shizgal

**Affiliations:** Center for Studies in Behavioral Neurobiology (CSBN)/Groupe de recherche en neurobiologie comportementale, Department of Psychology. Concordia University, 7141 Sherbrooke Street West, Science Pavilion, room #244, H4B 1R6 Montréal, QC, Canada

**Keywords:** Construction of resistant optical-fiber cables for behavioral optogenetics, Optogenetics, Optical fiber, Behavior

## Abstract

*In vivo* optogenetic experiments commonly employ long lengths of optical fiber to connect the light source (commonly a laser) to the optical fiber implants in the brain. Commercially available patch cords are expensive and break easily. Researchers have developed methods to build these cables in house for *invivo* experiments with rodents [1], [2], [3], [4]. However, the half-life of those patch cords is greatly reduced when they are used with behaving rats, which are strong enough to break the delicate cable tip and to bite through the optical fiber and furcation tubing. Based on [3] we have strengthened the patch-cord tip that connects to the optical implant, and we have incorporated multiple layers of shielding to produce more robust and resistant cladding. Here, we illustrate how to build these patch cords with FC or M3 connectors. However, the design can be adapted for use with other common optical-fiber connectors. We have saved time and money by using this design in our optical self-stimulation experiments with rats, which are commonly several months long and last four to eleven hours per session. The main advantages are:

• Long half-life.
• Resistant to moderate rodent bites.
• Suitable for long *in vivo* optogenetic experiments with large rodents.

## Graphical abstract

**Figure:**
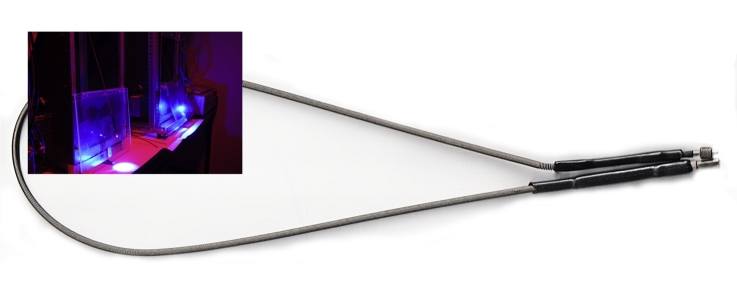

## Method details

The following steps illustrate how to build a robust optical patch-cord suitable for *in vivo* experiments with rats. This protocol complements other methods [1], [2], [3], [4] to build optical components for optogenetic experiments. Overall, we start with a 200 μm core multi-mode optical fiber of the desired length. We then attach the appropriate connector to be used with the behavioral testing setup (FC or M3 in this protocol). The length of the optical fiber is strengthened and protected by incorporating a layer of black shrink tubing and a stainless steel compression spring. We strengthen the opposite tip of the patch cord, which is attached to the optical implant on the animal’s head, by merging two stainless alloy ferrules and adding several layers of shrink tubing. This results in a strong and semi-rigid tip that serves as a holder and it is resistant to being dislodged when the animal moves around the setup.

Step 1: Before you begin, read this section completely and refer to Table 1 for a list of required materials. The assembling process takes sixty to ninety minutes. We prefer to use optical fibers with a smaller diameter core and/or smaller numerical aperture than that of the optical implant to which the patch-cord will be attached to. This is a preventive measure to avoid light loss. In principle, increasing the core diameter and/or the numerical aperture should retain light transmission in the face of small alignment errors. When two ferrules are joined by means of a ceramic sleeve, alignment may well vary somewhat as a function of the animal’s movements. Avoiding variation in light transmission is of particular importance in psychophysical experiments, which require tight, graded control of optical power. The following steps and inventory are appropriate to make a 200 μm core multi-mode 0.39 NA fiber patch-cord compatible with a 300 μm multimode implant, but they can be adapted for other core diameters, specially if experimenters are concerned about the larger tissue damage produced by using thicker fibers.

**Fig. 1:**
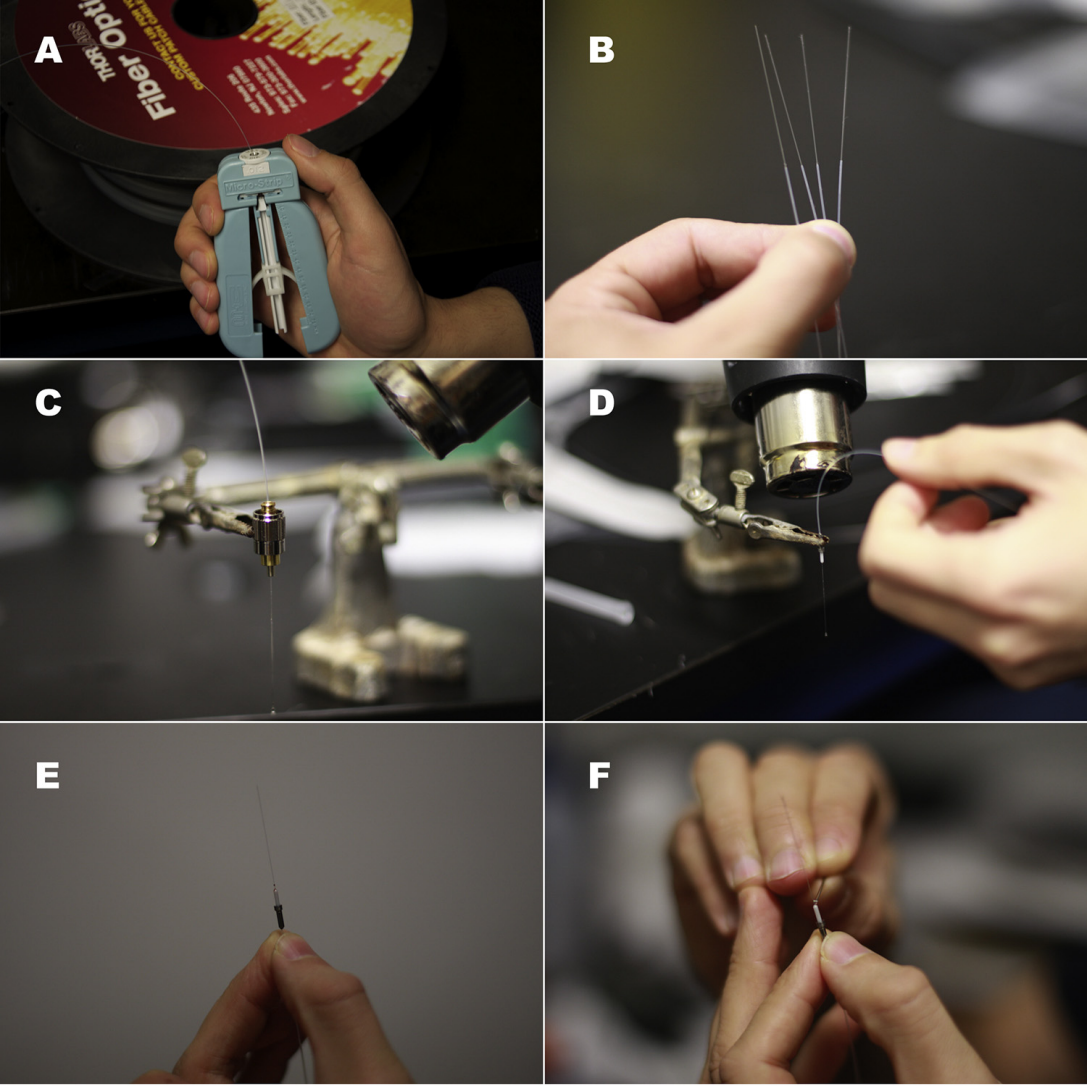
(A) Stripping 200 μm multimode optical fiber. (B) 35 mm of exposed optical fiber core in four optical fiber threads. (C) FC connector on third hand. Notice that the clamping occurs away from the tip of the connector. (D) Curing the resin inside the M3 connector. The process is similar for FC connectors. Warning: following this step the connectors will be very hot. (E) Cured resin at the tip of the M3 connector. The fiber should be glued to it rigidly and should cover the lumen. (F) Troubleshooting when there is no excess of resin at the tip of the connector. A very small amount of resin is applied to the lumen of the connector.

Step 2: Use the optical fiber stripper to remove the optical fiber buffer and expose 35 mm of the optical-fiber cladding (Fig. 1A). Measure the optical fiber from the unstripped edge (Fig. 1B) and cut it to the desired length using scissors or wire cutters.

Step 3: Mix the heat curable epoxy resin according to the manufacturer’s instructions, and load a 3 ml syringe with the mixed resin. Attach a 21 gauge blunt tip needle (Braintree Scientific, Inc., Braintree, MA) to the syringe.

Place the FC (Fiber Instruments Sales Inc., New York, USA) or M3 (Doric lenses, Québec, Québec, Canada) connector (Fig. 1C and D) on the third hand with the small lumen facing downwards. Fill the internal volume of the connector with resin. Insert the fiber core all the way through the connector, leave in place, and cure the resin with the heat gun until it darkens. Be careful not to overheat the epoxy: some optical fibers may be damaged by excessive heat, resulting in lower light transmission. Any resin spills on the connector should be wiped off before heat-curing the resin.

When the resin has cured, the tip of the M3 or FC connector should have a small excess drop of resin on the 240 μm lumen (Fig. 1E), which will hold the optical fiber core in place when the end of the fiber is polished. If the excess resin is not present, a 26 gauge needle or a piece of optical-fiber buffer can be used to apply a small drop of resin to the area (Fig. 1F).

**Fig. 2:**
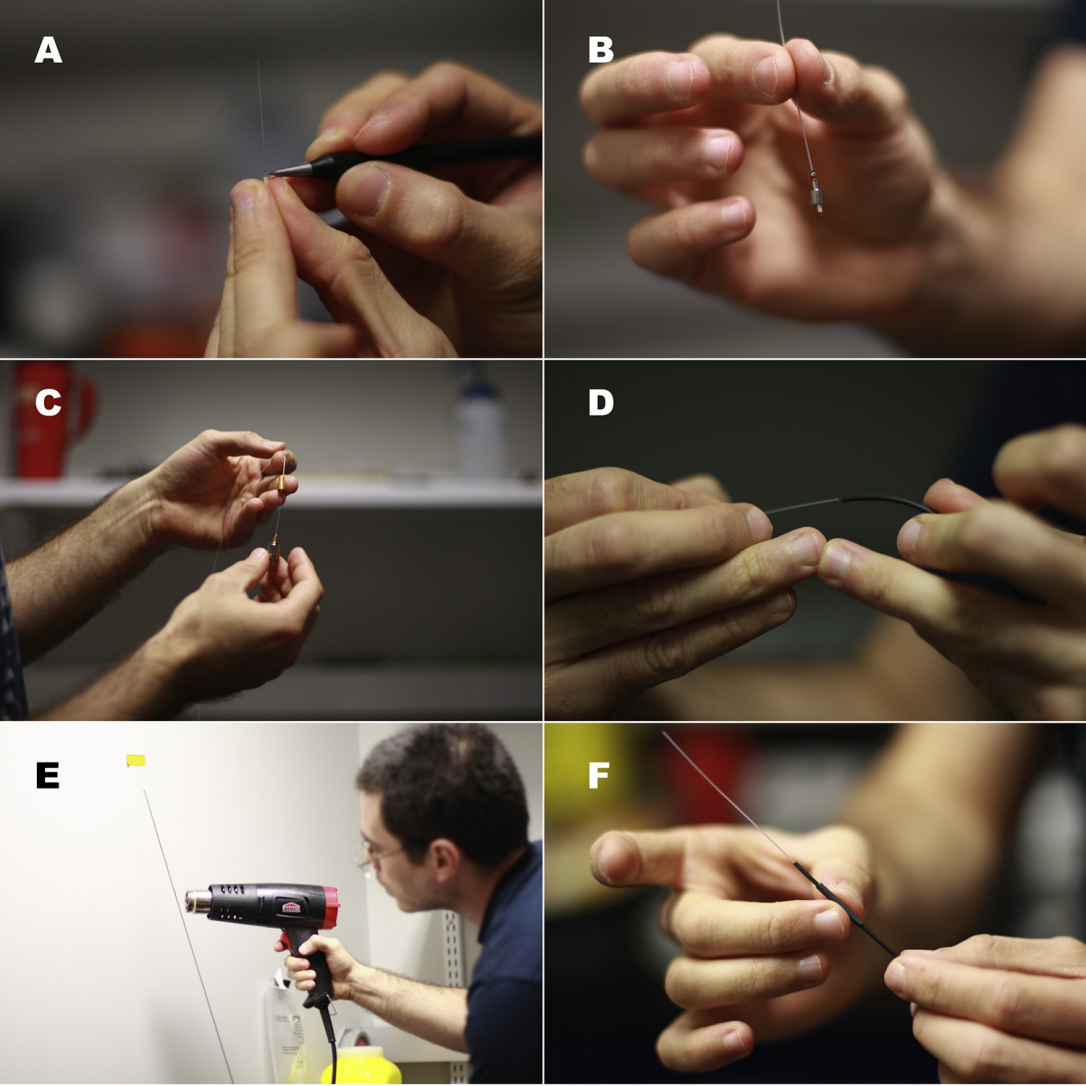
(A) Making a groove at the edge of the fiber. Notice that the diamond wedge scribe is kept horizontal. Do not break the optical fiber core doing this. (B) An M3 connector with the leveled cut and the bolt. (C) The boot of the FC connector is inserted. The boot should be crimped-on tightly using the specialized crimping tool. (D) Inserting the optical fiber through the 3/64′′ shrink tubing. (E) Shrinking the 3/64′′ shrink tubing tightly onto the fiber. Make sure it is even, “bumps” would not allow the stainless steel compression spring to fit through. (F) Insert the small shrink tubing provided with the M3 connector over the new cladding. Push it all the way through but do not shrink it yet.

Step 4: Use the diamond scribe to make a small straight groove in the side of the optical fiber core, right at the edge of the hardened resin on the tip of the connector (Fig. 2A). Flick-off the protruding length of optical fiber core to leave a level surface. Avoid breaking the core with the diamond scribe.

If working with the M3 connector, insert the internally threaded nut (provided by the manufacturer) until it reaches the connector (Fig. 2B).

If working with an FC connector, pass the optical fiber through the metal boot (Fig. 2C) and crimp it in place using the special-purpose tool (not shown).

Step 5: Cut a piece of 3/64′′ shrink tubing (approximately 10% shorter than the length of the optical fiber), and pass the optical fiber through it until it is in contact with the connector (Fig. 2D). Secure the fiber to a vertical surface, and with the connector facing downwards, apply heat to evenly and tightly shrink the tubing around the fiber (Fig. 2E). Make sure that the shrink tubing cladding is even; avoid having bumps on the tubing.

If working with M3 connectors, pass the small-bore shrink tubing (provided by the manufacturer, Fig. 2F) over the fiber until it protrudes slightly beyond the metallic end of the M3 connector. Do not shrink this tubing until the fiber tip has been properly polished (see Step 11).

If working with the FC connector, slide the rubber protector provided by the manufacturer over the metallic boot (not shown).

**Fig. 3:**
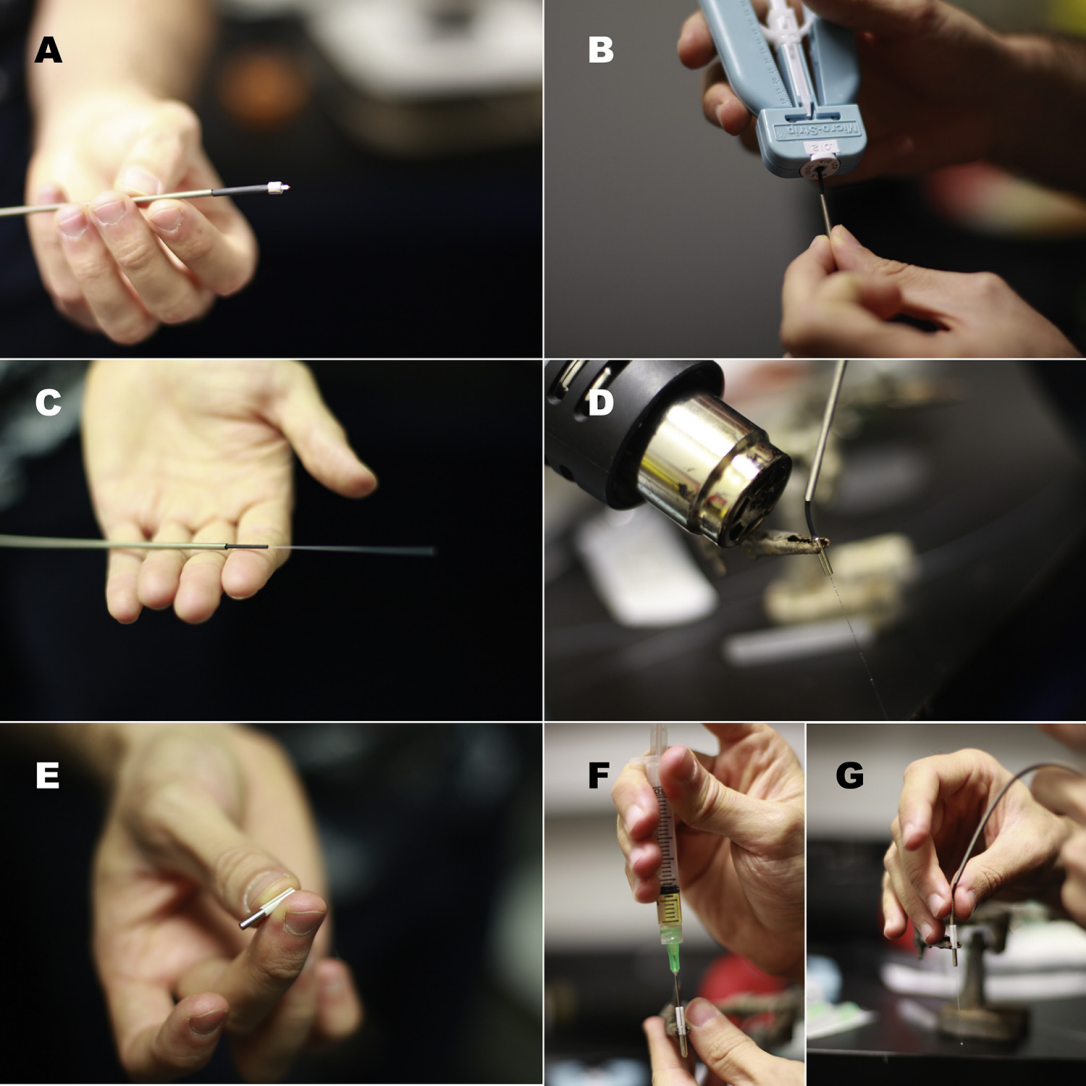
(A) An unfinished patch cord with the stainless steel compression spring and the small shrink tubing from the M3 connector inserted through it. (B) Striping the other end of the optical fiber. (C) The spring, shrink tubing, and exposed optical fiber cladding are shown. (D) The exposed optical-fiber core is passed through a ferrule loaded with resin. The drops of resin that accumulate on the core should be wiped off before heat-curing the resin. (E) A ceramic split sleeve is attached to the edge of a ferrule (on the side with the large opening). (F) The component shown in (E) is loaded with resin. A thin layer of resin should also cover the internal walls of the ceramic sleeve. (G) The optical fiber core shown in (D) is passed through the second ferrule until both ferrules are in direct contact.

Step 6: Pass the optical fiber through the stainless-steel compression steel spring (Fig. 3A), and strip 45–50 mm of the optical fiber beyond the connector (Fig. 3B). When stripping, avoid leaving a large gap of unstripped fiber between the shrink tubing and the exposed optical fiber cladding (a small gap, between 5 and 8 mm long, is acceptable, Fig. 3C).

Step 7: Place a stainless-steel ferrule in the third hand (the 240 μm drilled part should face downwards). Fill up the internal volume of the ferrule with heat-curable epoxy, and pass the long section of exposed fiber core all the way through until it cannot be pushed any further. It is very important to remove all traces of resin from the inserted optical-fiber core. This can be done using a delicate-task wipe and alcohol. Do not cure the resin inside the ferrule until certain that the fiber core is resin free. Apply heat to cure the resin and fix the ferrule in place, making sure to leave approximately 40 mm of optical fiber core protruding from the ferrule (Fig. 3D).

Step 8: Fasten a ceramic split sleeve to another ferrule, making sure the 240 μm lumen is facing away from the sleeve and that most of the ferrule remains exposed (*i.e.*, about 1/4 of the length of the ferrule should enter the ceramic sleeve, Fig. 3E). Attaching this second ferrule to the patch cord provides a rigid area to grip when attaching and detaching the cable. This also prevents the cable from bending sharply as the animal makes sudden movements and reduces the likelihood that contact with the levers or the chamber walls will detach, break, or dislodge the patch cord. In our hands, cables with single ferrules at the tip have a shorter half-life than those built using two ferrules in tandem.

Place the attached ferrule on the third hand and load it with heat curable epoxy (Fig. 3F). Allow for some resin to reach the inside of the ceramic sleeve. Insert the optical fiber core through the ferrule, until both ferrules are in contact with each other within the ceramic sleeve (Fig. 3G). Remove excess resin and heat cure the remainder in place.

When the resin has cured, a small excess should remain at the tip of the ferrule. If not present, gently apply a very small amount of resin to the area (as in Fig. 1F). Again, use the diamond scribe to make a groove on the side of the optical fiber core, at the edge of the cured resin. Make sure not to break the fiber when making the groove. Flick to cut the fiber at the level of the groove.

**Fig. 4:**
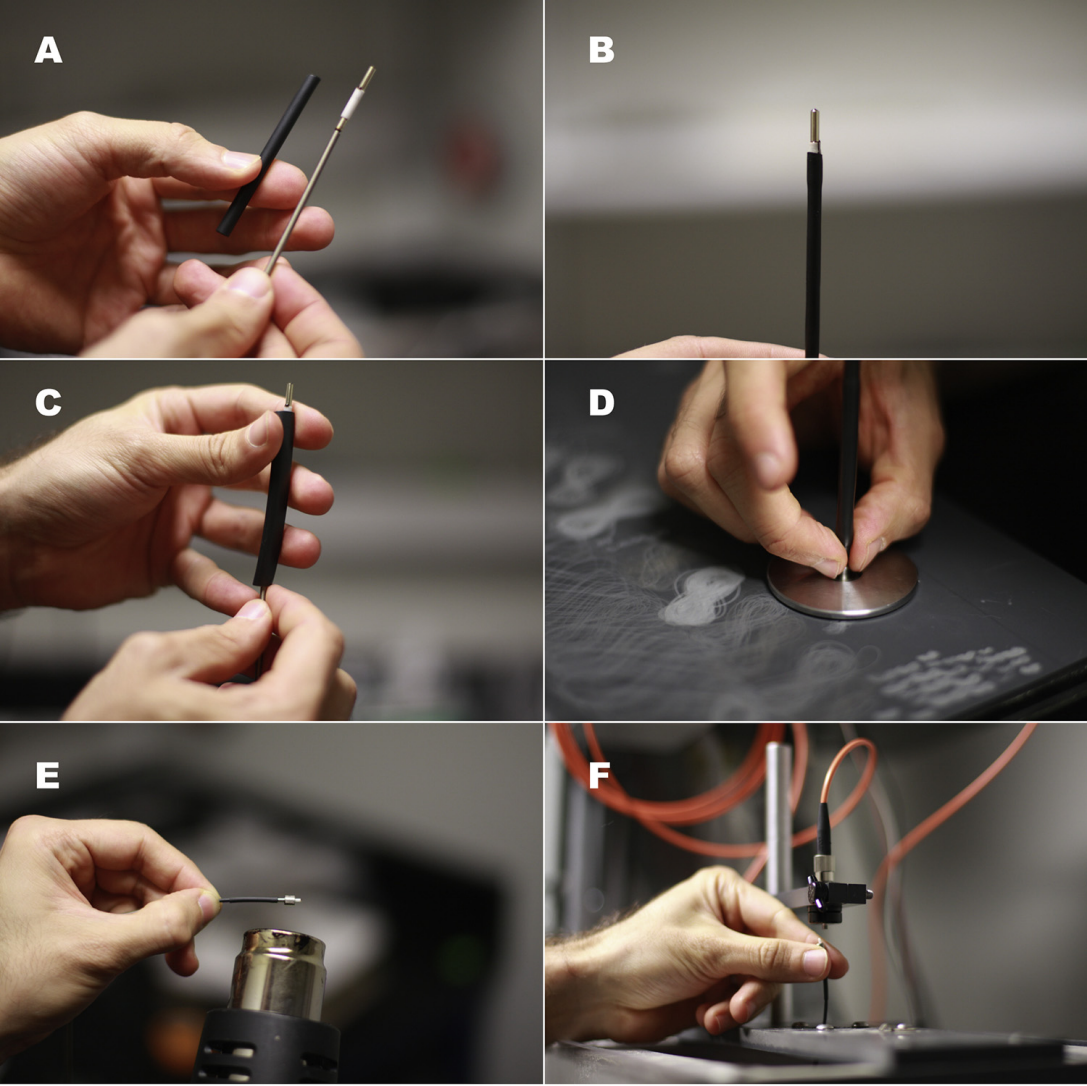
(A) A piece of adhesive shrink tubing is inserted through the tip of the patch cord, keeping the spring in contact with the ferrule. Note that the excess optical-fiber core has already been flicked off at this point, similarly to Fig. 2A. (B) The aspect of the semi-rigid tip when the adhesive shrink tubing has been fixed in place. (C) Adding an extra layer of protection and rigidity by inserting a piece of 1/4′′ shrink tubing. (D) Use the 5 μm silicon carbide polishing paper and disk to polish the cured resin off the tip. Notice the “eight shape” marks left on the paper. The polishing disk can also be used with FC connectors. (E) Shrink the short piece of shrink tubing provided with the M3 connector in place. Make sure to wrap it onto the metallic part of the connector and the covered optical fiber. Do not do this until the fiber has been properly polished: it may make visualizing through the 200× fiber scope more difficult. (F) Attaching the M3 connector to the corresponding 1 × 1 optical swivel (Doric lenses, Québec, Québec, Canada) for testing.

Step 9: Push the spring over the fiber until it is in contact with the ferrule. Insert the end with the attached ferrules through a 2” piece of the adhesive shrink tubing (Fig. 4A). Push the ferrules through the adhesive shrink tubing until the ferrule glued to the tip of the cord protrudes completely and some shrink tubing covers a section of the spring; the attached ceramic sleeve should be almost completely covered by the tubing. Heat-shrink the tubing in place, making sure the spring is in close contact with the ferrule. Apply light pressure around the tubing while it is still hot to produce tight contact with the spring and the ceramic sleeve below it (Be careful: the tubing can be very hot!). Some adhesive from the inside of the tubing may spill out. Avoid getting it on the exposed ferrule. This step stiffens the tip of the patch cord and secures the spring in place (Fig. 4B).

Step 10: Strengthen the tip by inserting it into a piece of 1/4′′ shrink tubing (approximately 2′′ long) and heat-shrinking this tubing over the already stiffened patch-cord tip (Fig. 4C), taking care to leave the ferrule exposed. This step can be repeated until the desired rigidity or external diameter is achieved. We recommend using at least one layer of 1/4′′ shrink tubing.

Step 11: Fibers need to be adequately polished to achieve optimal performance; instructions can be downloaded for free from the manufacturer’s web site: https://www.thorlabs.com/thorproduct.cfm?partnumber=FN96A. Polish the tip of the patch cord on the 5 μm silicon carbide polishing paper by repeatedly tracing a figure eight pattern with the exposed ferrule tip. Use the polishing puck to keep the ferrule vertical while polishing (Fig. 4D). Applying too much pressure when polishing can damage the optical-fiber core. Once the cured resin at the tip of the ferrule has been polished away, clean the tip of the fiber using isopropyl alcohol and a delicate-task wipe. Replace the silicon carbide paper with the 3 μm polishing paper and repeat the polishing process. It is helpful to keep this fine polishing paper clean between polishes by using isopropyl alcohol and delicate-task wipes. Lubricating the patch-cord tip with a drop of distilled water makes the polishing process easier. Repeat the process with progressively finer polishing paper (*i.e.*, 1 μm and 0.3 μm) until the polished optical fiber core appears circular, smooth, light gray, and without scratches when viewed through the 200× fiber scope. Repeat these steps to polish the FC connector.

If using an M3 connector, clamp a straight hemostat to the connector tip and keep it flat against the polishing paper [3]. Make sure the long axis of the tip of the connector is perpendicular to, and in touch with, the paper. Polish by drawing a series of figure eight on the silicon-carbide paper with the tip of the connector. The hemostat should help avoid uneven polishing. Continue the process on progressively finer paper, as you did to the other end of the patch cord. The optical fiber core should be circular, light gray, and glossy when viewed through the fiber scope. The small shrink tubing from the M3 connector should extend slightly over the metallic part of the connector so as to wrap around it and the optical fiber, while still allowing the threaded nut to rotate and move along the base of the connector (Fig. 4E).

**Fig. 5:**
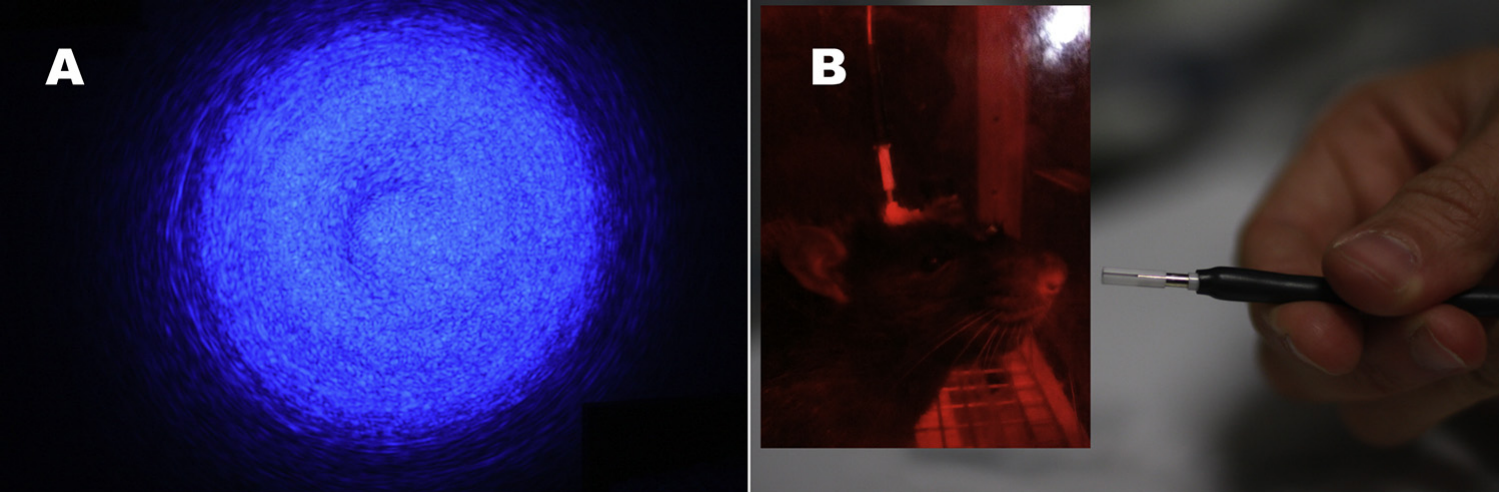
(A) visual inspection. The light coming out from the patch cord tip should be concentric. (B) A ceramic sleeve is attached halfway through the tip of the patch cord. Inset: the ferrule from the implanted fiber on the rat’s head should occupy the other half, keeping both ferrules in direct contact with each other.

Step 12: Test the patch cord in the behavioral apparatus. Attach the patch-cord connector to the optical rotary joint (Fig. 4F) or the corresponding light output of your setup. Carefully point the patch cord tip toward a vertical non-reflective surface and turn on the light source (Caution: do not use full laser power in this test, and wear appropriate protective goggles, except when you are certain that the beam is pointing away from you, and you wish to view the pattern it forms on the non-reflective surface). A properly polished patch cord will produce a pattern of closely spaced concentric rings (Fig. 5A). Confirm that the desired optical power is emitted by the patch cord by using the photodiode sensor and the optical-power meter. Adjust the laser power to determine the maximal light output from the cord. If the patch cord is properly built and polished, the light loss should not exceed 15% per connection (top and bottom). It is best to provide “headroom” by using patch cords that exceed the required output. Avoid using patch cords that cannot transmit at least 10% more power than required in the experiment.

Insert the metal ferrule at the patch-cord tip halfway into a ceramic sleeve (Fig. 5B). The metallic ferrule attached to the optical implant should fill the empty half of the ceramic sleeve so that both ferrules are in close contact inside the sleeve (Fig. 5B inset).

These patch cords have held up well for several months under heavy use in daily experimental sessions lasting from four to eleven hours. We have used them successfully with or without a counterbalancing arm (MED Associates Inc., St. Albans, VT. Product number: PHM-110-SAI). When cut to a length of 2.32 ft (71 cm) the patch cords weigh approximately 0.49 oz (14 g).

## Additional information

### Damage prevention and maintenance

Always handle the patch cord by the reinforced ferrule tip. This is particularly important when detaching it from the optical implant: pulling directly on the cord may damage it.

On rare occasions, the patch cord may detach from the rat’s head during an experimental session, and the rat may seize it and bite it. This can be prevented by routinely replacing the ceramic sleeve that connects the cord to the optical implant; these sleeves tend to loosen with continued use.

The metallic shielding and tubing layers tend to survive some nibbling by a rat. When this occurs, new layers of 1/4′′ shrink tubing can be used to re-strengthen the damaged part. In rare cases, a rat may become adept at detaching the patch cord, and may destroy it if given enough time. Thus, it is useful to apply a few drops of a quinine solution to the patch cord tip to dissuade the animal from biting.

After several months of usage, some loss of power may occur. This can be remedied by gently wiping the ferrule end with alcohol, or by re-polishing it if the problem persists.

## Figures and Tables

**Table 1:** Inventory.

Material	Company	Catalog no.	Quantity
200 μm, 0.39 NA multimode optical fiber	Thorlabs Inc., Newton, New Jersey, USA	FT200EMT	Cut to desired length
200 μm optical fiber stripper	Thorlabs Inc., Newton, New Jersey, USA	T12S21	1
Heat curable epoxy resin part A	Precision Fiber Products Inc., Silicon Valley, California, USA	PFP-353ND-16OZ-A	1 ml
Heat curable epoxy resin part B	Precision Fiber Products Inc., Silicon Valley, California, USA	PFP-353NC-16OZ-B	0.1 ml
M3-230 μm connector^a^	Doric Lenses Inc., QC, Canada	F210-0409	1
FC alloy connector drilled to 240 μm^b^	Fiber Instruments Sales Inc., New York, USA	30126G2-240	1
FC crimp tool^b^	Thorlabs Inc., Newton, New Jersey, USA	CT042	1
Diamond wedge scribe	Fiber Instruments Sales Inc., New York, USA	FO9OW	1
3/64′′ shrink tubing	Newark Element14. Palatine, IL, USA	84N583	∼10% shorter than fiber
1/4′′ shrink tubing	Newark Element14., Palatine, IL, USA	84N588	∼2′′
0.125′′ ID adhesive shrink tubing	Newark Element14., Palatine, IL, USA	48W4786	∼2′′
Stainless steel compression spring, 0.1′′ ED, 0.07′′ ID	Heilplex east end., Montreal, QC, Canada	Custom order	Cut to desired length
Stainless alloy ferrule drilled to 240 μm	Fiber Instruments Sales Inc., New York, USA	F10061F240	2
Ceramic split sleeve	Fiber Instruments Sales Inc., New York, USA	F18300SSC25	2
5 μm silicon carbide polishing paper	Thorlabs Inc., Newton, New Jersey, USA	LFG5P	1
3 μm aluminum oxide polishing paper	Thorlabs Inc., Newton, New Jersey, USA	LFG3P	1
1 μm aluminum oxide polishing paper	Thorlabs Inc., Newton, New Jersey, USA	LFG1P	1
0.3 μm aluminum oxide polishing paper	Thorlabs Inc., Newton, New Jersey, USA	LFG03P	1
Polishing puck	Thorlabs Inc., Newton, New Jersey, USA	D50-FC	1
Polishing pad and polishing plate	Thorlabs Inc., Newton, New Jersey, USA	NRS913A CTG913	1
200× fiber scope	Thorlabs Inc., Newton, New Jersey, USA	FS200	1
Digital power meter	Thorlabs Inc., Newton, New Jersey, USA	PM100D	1
Photodiode power sensor	Thorlabs Inc., Newton, New Jersey, USA	S121C	1
Threaded fiber adapter for sensor	Thorlabs Inc., Newton, New Jersey, USA	S120-FC	1

List of required materials and tools.

a The M3 connector is not necessary if working with the FC connector.

b These materials are not necessary if working with the M3 connector.
